# Supplementary material for: Is Promotion of Mobility in Older Patients Hospitalized for Medical Illness a Physician’s Job?—An Interview Study with Physicians in Denmark
Source: Geriatrics (Basel). 2020 Oct 10;5(4):74. doi: 10.3390/geriatrics5040074 (PMC7709691; doi:10.3390/geriatrics5040074)
Supplement: Supplementary file 1 [file geriatrics-05-00074-s001.pdf]

Appendix1. Interview guide for semi-structured interviews with physicians

Introduction: Start by presenting yourself. Tell about the study and the interview conditions:

> *"As part of the WALK- Copenhagen project we're in the process of developing a stakeholder-designed intervention aiming at getting older medical patients up and about and at increasing the number of daily steps by about 1000 steps during hospitalization and after discharge. The intervention, which has been designed in workshops with stakeholders, implies that you as a physician must prescribe walking within the first 24 hours after admission. This has to be done at rounds. The prescription will be on time spent walking per day. Since the intervention is conditioned by the participation of you as physicians, its very important that we get your input on the design of the intervention to assure that its useable in daily praxis. I expect the interview to be of approximately 30-45 minutes. I'll record the interview and when it's transcribed, we'll assure that nothing refers to you. You should know, that you can withdraw your consent at any time, should you wish to do so".*

Interview guide

Domain	Questions
Knowledge	- Have you heard about the WALK-Cph project? When?<br>- Do you know about the contents of the project?<br>- What's your opinion on the relevance of this project?<br>- Do you think it's important that older adults are mobile during hospitalization? Why/why not?
Skills	- To what extent do you, in your daily work as a physician, focus on how much older medical patients move (stand and walk) during hospitalization?<br>- In which situations? Are there any situations in which you think it's important that the older patient is mobile? Can you describe such a situation?

	- Do you talk to the patients about being mobile?<br>- As part of the WALK-Cph intervention, physicians must prescribe walking time? What's your opinion on this?<br>- Do you experience a lack of skills or competencies with regards to prescribing walking time?
Social/professional role and identity	- To what extent do you find it a physician's job to focus on getting patients up and about? Why/why not?<br>- Our observations from the field study in four medical departments, yours included, showed that, in general, physicians did not focus on how much the patients were up walking and standing. Why do you think that is?<br>- Do you, among physicians, discuss the older patients' mobility or lack thereof? Can you elaborate on these discussions?<br>- What do you think it takes to make physicians consider a focus on mobility their responsibility? Do you think the WALK-plan can support this responsibility?
Beliefs about capabilities	- Do you think you will and can fill out and sign the WALK-plans?<br>- What can prevent you from doing this?<br>- We observed that quite a few patients were not motivated to get out of bed. How do you think you can motivate these patients?
Optimism	- Do you think the intervention can support an enhanced focus on mobility?<br>- Where do you see the greatest barriers and facilitators?
Beliefs about consequences	- What do you think will be the consequence for the patients by prescribing walking time?<br>- What will be the consequences for the physicians?<br>- And for cross-professional collaboration?
Reinforcement	- What can we do to overcome possible barriers?
Intentions	(Asked above: Do you think you will fill out and sign the WALK-plans?)
Goals	- What does it take for this intervention to be successful?
Memory, attention and decision processes	- What does it take for you as physicians to fill out/prescribe and follow-up on the WALK-plans?<br>- Is there anything that we or others can help with?
Environmental context and resources	- What do you think of the WALK-path that will be put up? Is there anything we should be particularly aware of?
Social influences	- Is there anyone in particular (other health care professionals, specific persons, management) who has to support the intervention before you do?<br>- How important is it that your colleagues fill out the WALK-plans? Do no fill out the plans?
Emotion	No questions.
Behavioral regulation	- What will it take for you to change your behavior regarding patient mobility? If you're willing to a change?

	What is needed to make mobility more of a point of focus?
